# NIR-II-activated biocompatible hollow nanocarbons for cancer photothermal therapy

**DOI:** 10.1186/s12951-021-00884-7

**Published:** 2021-05-13

**Authors:** Zhourui Xu, Yinling Zhang, Weixiao Zhou, Lijian Wang, Gaixia Xu, Mingze Ma, Fenghua Liu, Zan Wang, Yucheng Wang, Tiantian Kong, Binyuan Zhao, Weiping Wu, Chengbin Yang

**Affiliations:** 1 Guangdong Key Laboratory for Biomedical Measurements and Ultrasound Imaging, School of Biomedical Engineering, Health Science Center, Shenzhen University, Shenzhen, 518060 China; 2 State Key Laboratory of Metal Matrix Composites, School of Materials Science and Engineering, Shanghai Jiao Tong University, Shanghai, 200240 China; 3 Laboratory of Thin Film Optics, Shanghai Institute of Optics and Fine Mechanics, Chinese Academy of Sciences, Shanghai, 201800 China; 4 School of Physics and Optoelectronic Engineering, Xidian University, Xi’an, 710071 China

**Keywords:** Photothermal therapy, NIR-II, Biocompatible, Hollow carbon nanospheres, Cancer treatment

## Abstract

Photothermal therapy has attracted extensive attentions in cancer treatment due to its precise spatial-temporal controllability, minimal invasiveness, and negligible side effects. However, two major deficiencies, unsatisfactory heat conversion efficiency and limited tissue penetration depth, hugely impeded its clinical application. In this work, hollow carbon nanosphere modified with polyethylene glycol-graft-polyethylenimine (HPP) was elaborately synthesized. The synthesized HPP owns outstanding physical properties as a photothermal agent, such as uniform core-shell structure, good biocompatibility and excellent heat conversion efficiency. Upon NIR-II laser irradiation, the intracellular HPP shows excellent photothermal activity towards cancer cell killing. In addition, depending on the large internal cavity of HPP, the extended biomedical application as drug carrier was also demonstrated. In general, the synthesized HPP holds a great potential in NIR-II laser-activated cancer photothermal therapy.

**Supplementary Information:**

The online version contains supplementary material available at 10.1186/s12951-021-00884-7.

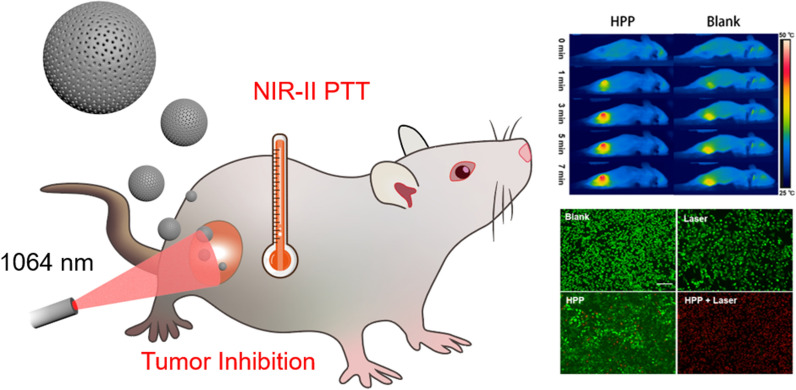

## Introduction

Cancer, one of the most dreadful diseases in the world, caused millions of human deaths every year [1]. In the past few decades, lots of investments have been devoted to developing therapeutic modalities, including chemotherapy[2], radiotherapy [3], and surgery [4]. Although these treatments have been proved to be effective in preclinical and clinical trials, most patients were still facing unsatisfactory treatment outcomes and high mortality, which were attributed to tumor heterogeneity, severe side effects, time-consuming processes, and slow therapeutic effect [1]. To broaden the library of cancer therapeutic strategies and address the underlying drawbacks in conventional modalities, it is urgent to develop precise and efficient therapeutic methods.

Photothermal therapy (PTT) using remote-controlled and power tunable near-infrared (NIR) laser to induce localized tumor ablation by photothermal agents (PTAs) has attracted huge attention in the field of cancer treatment [5]. Briefly, under the exposure of NIR irradiation, hyperthermia generated by PTAs can be applied to kill cancer cells while avoiding collateral damages to nontargeted tissue. Various types of PTAs, including gold nanoparticles [6–8], copper chalcogenide nanocrystals [9–11], organic nanoparticles [12], and two-dimensional (2D) materials [13], have been reported in previous studies. However, the therapeutic efficacy of PTT is usually subjected to the physical properties of PTAs, such as insufficient heat conversion efficiency (HCE), deteriorate photothermal response, and adverse side effects [14]. To maintain sufficient heat generation for efficient PTT, it is unavoidable to tradeoff between laser power-dependent phototoxicity and dosage-dependent dark toxicity. In addition, the efficacy of PTT was also affected by the penetration depth of the excited light sources. In recent years, a growing concern has been given to PTT in NIR-II (1000–1700 nm) optical window, owing to the reduced photon scattering, low tissue absorbance, high penetration depth, and high maximum permissible exposure (0.6 W/cm^2^) [15–17]. Yet the investigation of NIR-II PTAs is still at an infancy stage with only limited examples that have been demonstrated. NIR-II PTAs with high HCE and negligible cytotoxicity were rarely reported [18]. Therefore, developing safe and efficient NIR-II PTAs is highly desired for biological applications.

Over the past few years, nanocarbon materials emerged as promising functional probes in biological areas due to their limited cytotoxicity, low fabrication cost, stable optical properties and versatile surface modification [19, 20]. In addition, owing to their unique molecular structures, the sp^2^ domains in nanocarbon materials can efficiently absorb NIR light and excite surface plasmons, followed by converting the transmitting random dipoles and resonance into thermal energy [21]. The strong absorption in NIR regions and lower energy requirement for photo induction endows great potential in PTTs. Various nanocarbon materials, including nanocarbon suspensions [21], carbon nanotubes [22], graphene and graphene oxide [23, 24], have been reported for photothermal tumor ablation. Despite the promising features of nanocarbon materials, two major issues severely hinder the further development of them in clinical. First, most of the nanocarbon materials possess distinct batch-to-batch differences, which seriously undermine their reliability in cancer treatment [25]. Secondly, most of nanocarbon materials only respond to NIR-I light, which could not afford deep tissue therapy [26, 27]. Therefore, there is much room for the development of nanocarbon materials as PTAs with better performances in NIR-II window.

Considering the aforementioned aspects, we rationally designed and fabricated hollow carbon nanospheres as NIR-II responsive PTAs. Due to its uniform morphologies, stable structure and distinct absorption in NIR-II region, a remarkable high HCE of 45.1% was achieved under the irradiation of 1064 nm laser. In addition, with surface modification of polyethylene glycol-graft-polyethylenimine (PEG-g-PEI) polymer, the biocompatibility and aqueous dispersity were ensured. Hollow carbon nanospheres capped with PEG-g-PEI (termed as HPP) were further performed in vitro and in vivo experiments under a safe laser power density of 0.6 W/cm^2^. The results demonstrated limited cytotoxicity of HPP and precise and efficient therapeutic outcomes in the xenograft 4T1 tumor-bearing mice model. In addition, the porous carbon shell and large inner cavity of HPP can be further exploited as drug reservoirs. Nearly 60% of drug loading efficiency was achieved with the optimal condition. Therefore, HPP with unique structural properties and strong photothermal effects in NIR-II window possess great potential in precise and efficient cancer therapy.

## Materials and methods

### Chemicals and materials

TEOS, resorcinol, EDA and 10% hydrofluoric acid solution were purchased from Alfa Aesar, formaldehyde and ethanol were supplied by Sinopharm Chemical Reagent Co., Ltd. Doxorubicin (Dox) was purchased from Aladdin Reagent Co., Ltd. PEG-g-PEI was purchased from Sigma-Aldrich Co., Ltd. All chemicals were used without further purification.

### Preparation of PEG-g-PEI capped hollow carbon nanospheres

Hollow carbon nanospheres (HCNs) were fabricated according to our previous experience [28]. Briefly, silicon dioxide (SiO_2_) nanoparticles were synthesized with a modified Stöber process and were employed as hard templates [29]. To prepare the HCNs, 100 milligrams of SiO_2_ nanoparticles were thoroughly dispersed in a premixed solution composed of 6 mL ethanol and 14 mL Deionized (DI) water. Next, 20 mg of resorcinol, EDA and 30 µL of formaldehyde were added to the above solution sequentially at intervals of 5 min under vigorous stirring at 35 °C. After 10 min, 45 µL of TEOS (dispersed in 400 µL of ethanol) was further added into the system under vigorous stirring at 35 °C for 24 h. The solid compound was extracted by centrifugation with 8000 rpm for 5 min and dried at 60 °C overnight. The precursor product was then carbonized under nitrogen (N_2_) gas at 900 °C for 3 h. Then the hollow structures of HCNs were obtained after the removal of silica by etching with 10% hydrofluoric (HF) acid solution. The surface modification of HCNs was realized by mixing HCNs suspension and PEG-g-PEI solution overnight followed by centrifugation and re-disperse in PBS solution.

### Characterization of HPP

The transmission electron microscopy (TEM) micrograph was obtained using a HT7700 (Hitachi, Ltd) transmission electron microscope. The scanning electron microscopy (SEM) micrograph was obtained using a THERMO APREO S scanning electron microscope (Thermo Scientific Co., Ltd). The UV-Vis-NIR absorption spectrum was measured on a TP-720 spectrometer (Tianjin Tuopu Instrument Co., Ltd). The photothermal performances of the samples were evaluated by NIR laser (Changchun Leishi Photo-Electric Technology Co., Ltd) and FLIR A300 infrared thermal imaging camera (FLIR Systems Co., Ltd). The hydrodynamic size of the sample was measure by Zetasizer-Nano-ZS-90 dynamic light scattering device (Malvern Panalytical, Ltd).

### The measurement of photothermal effect

To measure the photothermal effect of HPP, the HPP suspension with a certain concentration was added into a plastic centrifuge tube. The tube containing HPP suspension was exposed to the NIR irradiation with different power densities. The temperature variation was recorded by an infrared thermal imaging camera. The photothermal stability of HPP suspension was measured by a continuous cycle (four times) of irradiation and natural cooling.

### The photothermal conversion efficiency

To evaluate the photothermal capability of HPP suspension, HPP suspension was irradiated by NIR laser until a saturation temperature was obtained and then cooled down to room temperature with the laser turned off. The photothermal conversion efficiency (*η*) can be calculated according to Eq. (1)

1 $${\eta} = {\frac{\text{hS}({T}_{max}-{T}_{ssur})-{Q}_{dis}}{\text{I}(1-{10}^{-{A}_{1064}})}}$$

The ${T}_{max}$ denoted as the saturation temperature of the irradiated sample, ${T}_{ssur}$ is the surrounding temperature. The ${Q}_{dis}$ means the heat loss from the light absorbed by the container. $\text{I}$ corresponding to the incident laser power. ${A}_{1064}$ is the absorbance of sample suspension at 1064 nm. Where $\text{h}$ represents the heat transfer coefficient and $\text{S}$ represents the surface area of the container. The value of $\text{hS}$ is calculated by using the following Eq. (2)

2 $${\tau }_{s} = \frac{{m}_{D}{c}_{D}}{hS}$$

${\tau }_{s}$ is the sample system time constant of sample suspension, ${m}_{D}$ and ${c}_{D}$ are the mass and heat capacity of the solvent. The ${\tau }_{s}$ can be calculated based Eqs. (3) and (4.)

3 $$\text{t}=-{\tau }_{S}ln\theta$$

4 $${\uptheta }=\frac{T-{T}_{surr}}{{T}_{max}-{T}_{surr}}$$

${T}_{max}$ is the steady-state temperature of photothermal agents while ${T}_{ssur}$ is the surrounding temperature.

### Drug loading

In order to load DOX into HPP, free DOX solution (40 µg/mL) and HPP suspension (varied concentration) were mixed under stirring overnight at room temperature. The DOX-loaded HPP were separated by centrifugation (8000 rpm, 5 min) and washed several times until the supernatant was colorless. The un-loaded DOX was calculated according to the standard cure of DOX and the absorbance of the supernatant sample at λ = 480 nm. The Dox loading efficiency (LE) can be calculated by Eq. (3)

5 $$LE\left(\%\right)=\frac{{m}_{oriDox}-{m}_{supDox}}{{m}_{HCNs}+{m}_{oriDox}}\times 100\%$$

where ${m}_{oriDox}$ is the mass of the original input DOX, ${m}_{supDox}$ is the mass of Dox in the supernatant and ${m}_{HCNs}$ is the mass of HPP.

### The cytotoxicity of HPP

In vitro cytotoxicity of HPP was evaluated by using 4T1 cells and MCF-7 cells. 4T1 cells were seeded in 96-well plates at a density of 5000 cells per well and grown in 5% CO_2_ at 37 °C overnight. Then, HPP with different concentrations were added into the medium and the cells were incubated for 24 h. At the end of incubation, 10 µL of 3-[4,5-dimethylthiazol-2-yl]-2,5-diphenyltetrazolium bromide (MTT) solution was added into each well and incubated for another 4 h. The supernatant in each well was aspirated and 150 µL of dimethyl sulfoxide (DMSO) was added to each well before the plate was examined using a microplate reader (Bio-tek, Epoch-2) at the wavelength of 490 nm. To evaluate the PTT efficacy of HPP, 4T1 cells (5000 per well) were seeded in each well of a 96-well plate and incubated overnight. Then, different concentrations of HPP were added to the medium. The cells were incubated in 5% CO_2_ at 37 °C for 4 h. After replacement of fresh medium, the cell samples were irradiated under 1064 nm laser of 0.6 W cm^− 2^ for 7 min. After that, the cells were further cultured for 24 h. The MTT process was carried out to assess the cell viability.

### Live and dead cell staining

In order to evaluate and visualize the PTT effect on treated cell samples. Live/Dead Cell Double Staining Kit was applied on cell samples. Briefly, 2 uL working solution of Calcein-AM and PI were added into each confocal dish. After incubated for 15 min and replaced with fresh culture medium, the cell samples were imaged by fluorescent microscope. Calcein-AM is a cell-permeable and non-fluorescent compound, while it will emit green fluorescence once enters into metabolically active cells. PI is a fluorescent nucleic acid stain that can permeate only the damaged membranes.

### Hemolysis Assay

The impact of HPP surface chemistry on red blood cells (RBCs) of mice and the influence of the protein corona interaction were evaluated by the standard hemolysis assays. A volume of 4 mL of blood was added to the anticoagulant tube. The blood was mixed gently and centrifuged at 3000 rpm for 20 min. The supernatant was discarded, and RBCs were washed a few times by suspending them in a PBS solution (pH 7.4). The final working suspension used for the hemolysis assay consisted of 5% (v/v) of RCBs in a PBS solution. To evaluate the hemolytic effect, HPP with different concentrations were incubated with RBCs (200 µL of a 5% suspension) for 2 h through a static method after gentle homogenization. The final volume of the hemolysis assay in all experiments was 1.0 mL. After incubation, the samples were further centrifugated and 100 µL of supernatants were extracted for quantification of hemoglobin by recording the absorbance at 540 nm. DI water and PBS solution were added into the RBCs samples as positive control and negative control respectively. The percentage of hemolysis was calculated based on Eq. (4),

6 $${\text{Hemolysis}}\;{\text{Rate}}\left( \% \right) = \frac{{ABS_{{sample}} - ABS_{{neg - contl}} }}{{ABS_{{posit - contl}} - ABS_{{neg - contl}} }} \times 100\%$$

where ${ABS}_{sample}$, ${ABS}_{neg-contl}$, ${ABS}_{posit-contl}$ are the optical density of tested samples, negative control and positive control, respectively.

### In vivo PTT therapy

The tumor-suppressive effects of HPP with or without 1064 nm laser irradiation were evaluated on Balb/c mice (~ 20 g), which were purchased from Guangdong Medical Laboratory Animal Center (No. 44007200082293). All animal experiments conform to the guidelines of the University Animal Care and Use Committee. The right axilla of each mouse was injected with 4T1 cells (murine hepatocarcinoma cell line, 1 × 10^6^ cells) subcutaneously to establish tumors. The tumors were allowed to grow for seven days to reach a size of around 100 mm^3^. Then, the 4T1 bearing Balb/c mice were randomly divided into 4 groups (PBS, PBS + laser, HPP, and HPP + laser). The intratumor injected HPP dose in 100 µL of PBS was 6 mg·kg^− 1^ body weight in total. Two groups as the treatment groups injected with PBS and HPP were irradiated with 1064 nm laser (0.6 W cm^− 2^) for 7 min. The other two groups were the control groups without irradiation. All the groups received twice injection and photothermal treatment on day 1 and day 3. The body weights and tumor volumes were monitored every two days. The tumor volumes were calculated using the following equation: Tumor Volume (V) = L× W^2^/2 (W is the shortest dimension. L is the longest dimension). Moreover, the tumor tissues in each group were harvested from mice 24 h after the first treatment were also dissected and fixed in paraformaldehyde used for hematoxylin and eosin (H&E) staining assay.

### Statistical analysis

All of the data are presented as mean ± standard deviation (SD). Statistical analyses were conducted by the Student’s t test to compare the results between two groups. The difference is regarded as statistical significance when *P* < 0.01, indicating with *.

## Result and discussion

### Synthesis and characterization of HPP

**Fig. 1:**
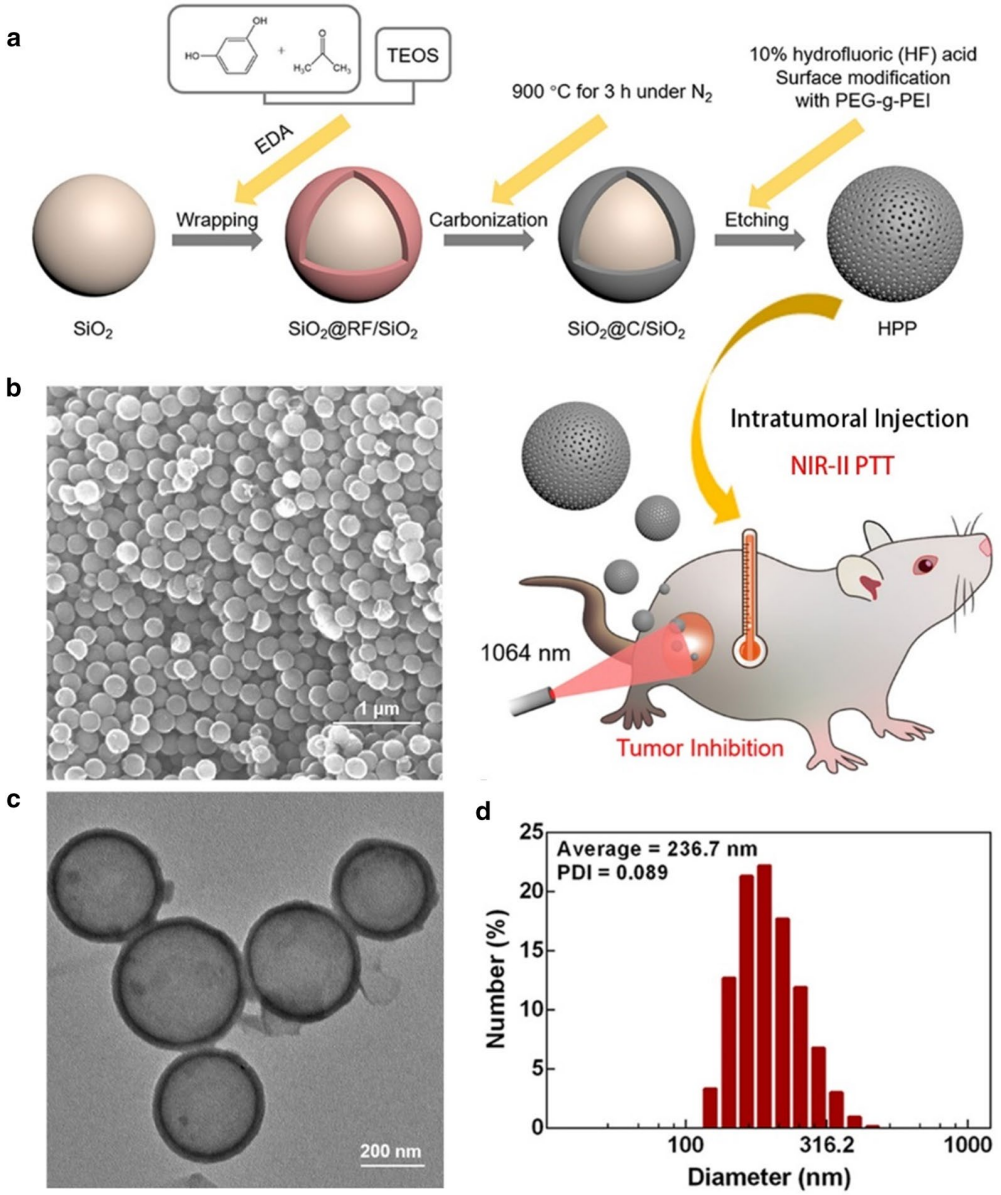
Synthesis and characterization of HPP. **a** Schematic illustration of the preparation processes of HPP and its application for cancer photothermal therapy. **b** SEM image of HPP; **c** TEM image of HPP; **d** Hydrodynamic diameter distribution of HPP

The research map and preparation process of HPP are illustrated in Fig. 1a. Monodispersed SiO_2_ nanoparticles with a diameter of ~ 180 nm was first applied as a hard template, followed by wrapping with a layer of resorcinol-formaldehyde polymer. Ethylenediamine (EDA) was used as the catalyst for polymerization and tetraethyl orthosilicate (TEOS) hydrolysis. After the carbonization and etching process, the inner SiO_2_ nanospheres as well as small SiO_2_ nanoparticles doped in the carbon layer were removed and left hollow carbon nanosphere. The morphology and size distribution of HPP sample were evaluated by SEM, TEM and Dynamic Light Scattering (DLS). The SEM image shows a highly uniform HPP with average diameters of 220 nm (Fig. 1b). After the inner SiO_2_ core was removed by etching, the hollow structure of HPP was obtained. The TEM image in Fig. 1c indicates that HPP possess a large inner cavity (~ 200 nm in diameter) with a shell layer of 15 nm. To maintain good dispersity and biocompatibility for biological applications, PEG-g-PEI polymer was covered on hollow carbon nanospheres. The hydrodynamic size and zeta potential of HPP in PBS solution was measured by DLS. As shown in Fig. 1d, the average diameter size of 236.7 nm with the Power-Distance Index (PDI) of 0.089 was confirmed. Such a size distribution was similar to the result obtained from TEM and SEM characterizations. In addition, after modification with PEG-g-PEI, the zeta potential of HPP reached + 34 mV, which is much higher than the zeta potential of naked HCN (+ 22 mV, Additional file 1: Fig. S1). Such a high positive zeta potential is beneficial for cellular uptakes due to the electrostatic interaction with negative charged cell membranes [30, 31].

### Photothermal effect of HPP irradiated by NIR-II laser

**Fig. 2:**
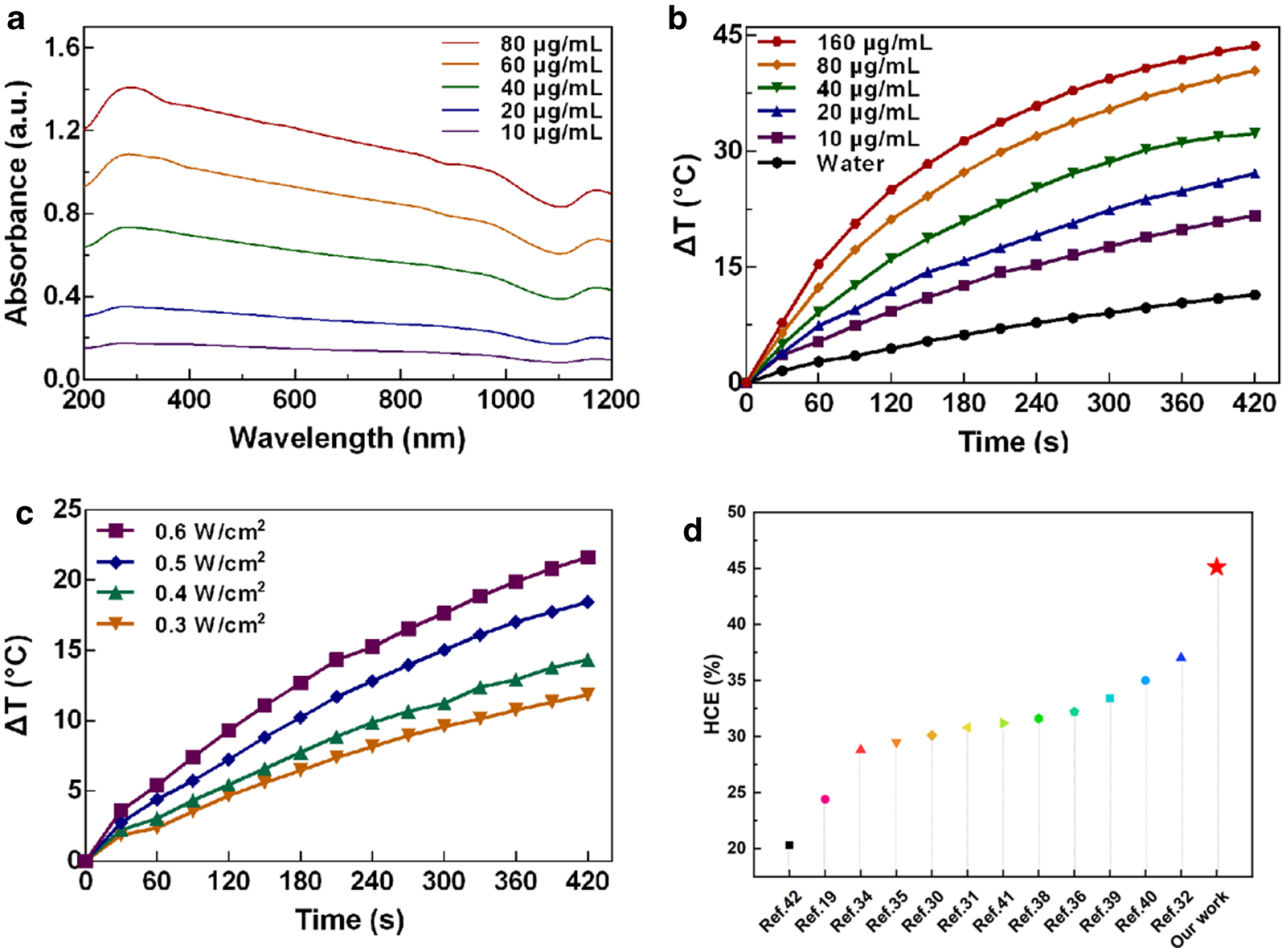
Optical and photothermal response of HPP. **a** Absorption spectra of HPP with different concentrations. **b** Photothermal response of HPP with different concentrations under 1064 nm laser exposure. **c** Photothermal response of HPP of 10 µg/mL under different laser power densities. **d** Comparison of HCE of HPP with previous reported NIR-II PTAs

The optical properties of the aqueous dispersion containing HPP were examined by the UV-vis-NIR spectroscopy. In Fig. 2a, the HPP exhibit a broad absorption covering the range of 200 nm to 1100 nm. The concentration dependent absorbance spectrum in Fig. 2a indicates the good dispersity of HPP. Motivated by the intense optical absorption in NIR region, the photothermal response of HPP was further evaluated under the irradiation of 1064 nm NIR-II laser. In Fig. 2b, the temperature elevation of pure water (as a reference) and HPP aqueous dispersion with different concentrations under laser exposure are shown. With a laser power density of 0.6 W/cm^2^ and the HPP concentration varied from 10 to 160 µg/mL, the temperature of the aqueous dispersions increased from 17 ℃ to 44 ℃ within 7 min. However, the pure water only increased 8 ℃ under the same conditions. Under laser irradiation, the temperature change profile in different concentrations of HPP were shown in Additional file 1: Fig. S2. The result indicates that the range of temperature increase was positively correlated with the concentration of HPP. In addition, by changing the laser power density from 0.3 to 0.6 W/cm^2^, HPP with the lowest concentration 10 µg/mL exhibited temperature increments from 11 to 21 ℃ (Fig. 2c). A linear relationship between temperature increment and laser power density was also observed (Additional file 1: Fig. S3). These results indicated that HPP can rapidly and efficiently convert NIR energy into thermal energy. To examine the corresponding HCE, 1 mL of HPP (40 µg/mL) was exposed to the 1064 nm laser until the maximum temperature was obtained followed by natural cooling with laser turning off (Additional file 1: Fig. S4a). According to the Eqs. (1–4), the HCE value of HPP is 45.1%, which is among the best of previous reported results on the NIR-II PTAs [18, 32–44] (Fig. 2d). The photostability of HPP was evaluated by repeating the lasering and cooling cycles several times. As shown in Additional file 1: Fig. S4b, the photothermal property is well maintained without deteriorating the maximum reachable temperature. Such result of HCE is much higher than that of gold nanorod (maxima absorbance at 1060 nm), a commercial PTA for research and pre-clinical studies. Under the same experimental parameters, the HCE of gold nanorods was only ~ 33% (Additional file 1: Fig. S5).

The morphology of HPP after laser irradiation was also examined by TEM. The results indicates that the size, shape, and dispersity of HPP was not influenced by laser irradiation (Additional file 1: Fig. S6). In conventional PTT, it is inevitable to make compromise between the laser power density and the concentration of PTAs. However, collateral damages to normal tissues by high power laser and long-term toxic side effects by over-dosage PTAs severely undermined the potential of PTT. HPP demonstrated herein can generate sufficient heat under a safe power density of 0.6 W/cm^2^ at a low concentration of 10 µg/mL, indicating that the material is an ideal candidate for safe and effective PTT.

### Biocompatibility analysis of HPP

**Fig. 3:**
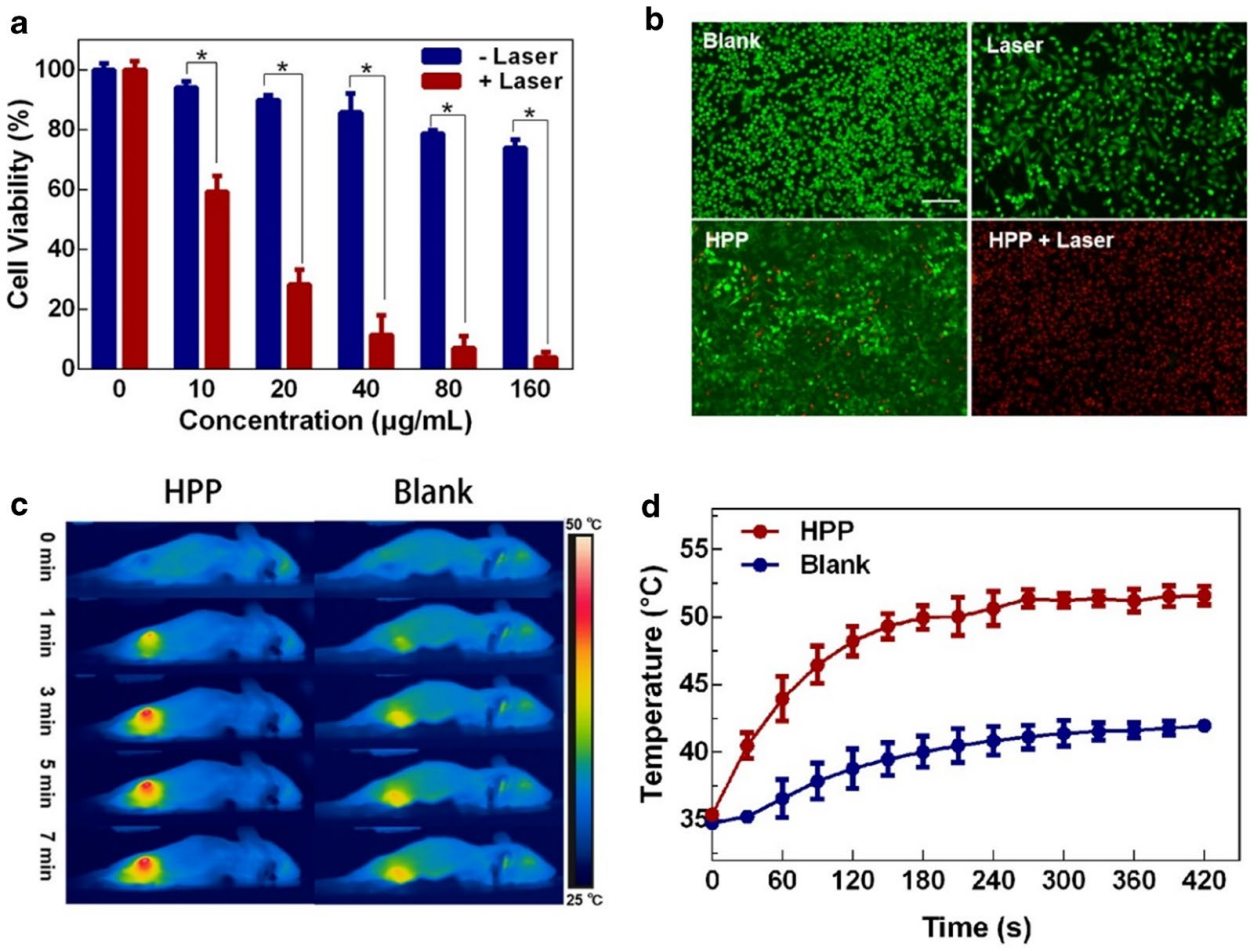
The PTT effects was evaluated in vitro and in vivo. **a** 4T1 cells viability after incubation with HPP with or without 1064 nm laser. **b** Live and dead staining of 4T1 cells treated under different conditions (Scale bar = 200 μm). **c** In vivo IR thermal images of 4T1 tumor-bearing mice intratumorally injected with PBS and HPP dispersion activated by 0.6 W/cm^2^ 1064 nm laser for 7 min. **d** Temperature increased profile of tumor area under laser irradiation. *p < 0.01

The ideal PTAs should not only have impressing ability in photothermal conversion but also are nontoxic or low toxic for biological applications. To evaluate the cytotoxicity of HPP, MTT assay with MCF-7 cells and 4T1 cells were used. As seen from Fig. 3a and Additional file 1: Fig. S7, after 24 h of incubation, more than 80% of cancer cells are alive with the concentration of HPP dispersion less than 80 µg/mL. Hemolysis rate is another important criterion to evaluate the biocompatibility of biomaterials. As shown in Additional file 1: Fig. S8, negligible hemolysis is observed within a large concentration range covering from 0 to 1600 µg/mL. These results indicated the limited cytotoxicity of HPP, especially with a concentration less than 80 µg/mL.

### Photothermal therapeutic effect in vitro and in vivo

The photothermal therapeutic effect of HPP was further investigated on 4T1 cells by applying a 1064 nm laser with 0.6 W/cm^2^. As shown in Fig. 3a, compared with un-irradiated groups, the laser treated cell samples exhibited distinct cell death. Nearly 40%, 70%, 85%, 90 and 95% of cell death were occurred by HPP with concentration of 10, 20, 40, 80 and 160 µg/mL, respectively. Hence, considering the biosafety issue and photothermal effects, HPP with a concentration of 40 µg/mL was selected in the following experiments. To better visualize the feasibility of PTT accomplished by HPP and 1064 nm laser, live (Calcein-AM, green fluorescence) and dead (PI, red fluorescence) staining experiments on treated 4T1 cells were performed. As shown in Fig. 3b, **4T1** cells without laser irradiation show high cell viability and no dead cells were observed. In contrast, in the case of HPP and laser co-treated group, almost all the cancer cells were eradicated. To evaluate in vivo anti-cancer effect, the therapeutic efficacy of HPP (40 µg/mL) on 4T1 tumor-bearing mice was studied under 1064 nm laser (0.6 W/cm^2^) irradiation. 100 µL of HPP aqueous dispersion was injected intratumorally prior to the laser irradiation. During the laser treatment, the temperature distribution of mice was recorded by IR thermal camera. As seen from Fig. 3c and d, the temperature in the tumor area of the HPP-treated group increased rapidly to 50 ℃ in 7 min. For the mice injected with PBS as control group, the tumor zone temperature only had a slight increment under the same condition.

### Anti-cancer effect in vivo

**Fig. 4:**
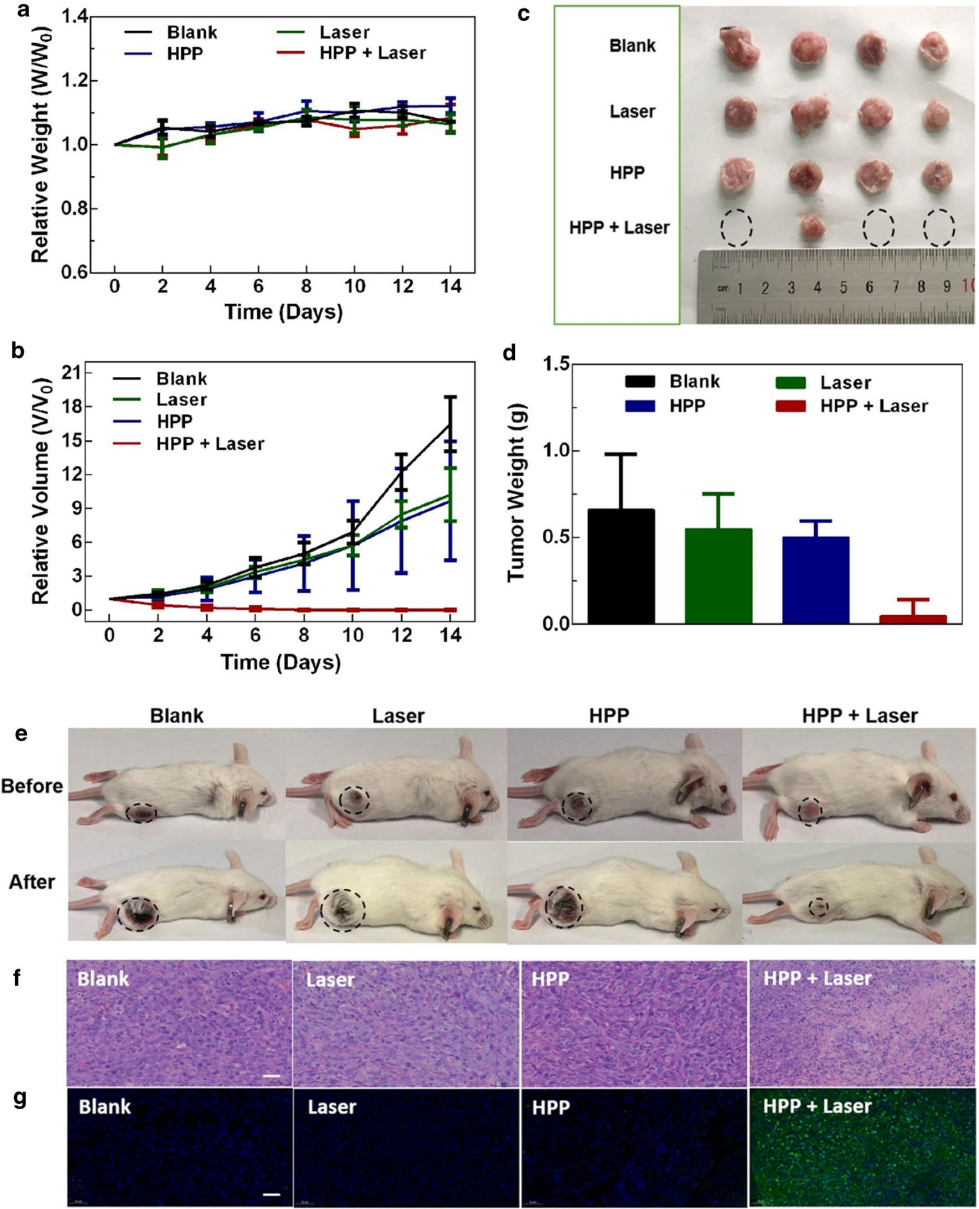
In vivo experiment, four groups of mice were treated with PBS, laser, HPP, and HPP combined with laser, respectively. **a** Average body weight of tumor-bearing mice in each group during the treatment periods. **b** Relative tumor volumes (compared to the whole-body volume) of tumor-bearing mice in each group. **c** Representative photograph of excised tumors from euthanized mice. **d** Average tumor weight of treated mice in each group. **e** Representative photographs of tumor-bearing mice after different treatments (Scale bar = 200 μm). **f** H&E and **g** TUNEL staining imaging of the tumor sections after photothermal therapy (Scale bar is 100 μm). *p < 0.01 vs. other groups

To investigate the anti-cancer effect of HPP, healthy Balb/c mice were inoculated subcutaneously with 4T1 cells on their right lower limb. When the tumor volume reached about 100 mm^3^, the tumor-bearing mice were used to study the anti-cancer efficacy of HPP in vivo. Twenty tumor-bearing mice were randomly separated into 4 groups (n = 5) and then intratumorally injected two groups with PBS and two groups with HPP. These four groups were further administrated with or without 1064 nm laser exposure at day 0. The body weight and tumor volume of each mouse were recorded every 2 days. Figure 4a and b show the change profiles of the mean body weights and average tumor volumes along with the treatment time. According to the results, the group treated with HPP combined with 1064 nm laser irradiation had no obvious tumor growth. In contrast, significant tumor growth was observed in other groups. Furthermore, no weight loss was detected in all groups during the course of treatment, which responded to the safety concerns of HPP-induced PTT.

After the observations over 14 days, all the mice were sacrificed, and their tumors were extracted. As shown in Fig. 4c, the tumor size in the group treated with HPP combined with 1064 nm laser irradiation is significantly diminished with three of them completely disappeared. However, without the photothermal effect, the tumor in the other three groups exhibited similar size. The histogram of the tumor volume of treated mice is further shown in Fig. 4d. Similar to the results acquiring from Fig. 4c, the HPP combined with 1064 nm laser demonstrated the strongest efficacy with the least tumor weight. As displayed in Fig. 4e, after 14 days, the tumor size in the group treated with HPP combined with 1064 nm laser irradiation is significantly inhibited. To further evaluate photothermal therapeutic effect in vivo, the histological examination of tumors was studied by hematoxylin and eosin (H&E) staining method. As shown in Fig. 4f, no significant changes in cell states were found in control group, NIR group and HPP group. The arrangement of tumor cells was loose and disordered. Conversely, the group treated with HPP combined with 1064 nm laser showed massive cell death in the tumor section. The morphology of cells was significantly altered, including karyopyknosis, karyorrhexis and karyolysis, which suggested that the tumor cells were heavily damaged. The cell apoptosis was further evaluated by TUNEL staining. The result indicated that the severe cell apoptosis of tumor cell was observed in HPP plus laser co-treated group, while no obvious apoptosis was observed in other groups (Fig. 4 g). In addition, the H&E staining was further performed pathologic analysis on the main organs (heart, liver, spleen, lung, kidney) of treated mice (Additional file 1: Fig. S9). No obvious lesions and organic injuries were detected in these pathological sections. Together, these results robustly demonstrated therapeutic strategy based on HPP and laser not only has great anti-cancer effect, but also has excellent biological safety in vivo.

In general, a new HPP with excellent photothermal conversion ability and limited bio-toxicity exhibited superior efficacy in cancer treatment has a great potential as NIR-II PTAs. Moreover, apart from the superior photothermal effect, the large inner cavity and mesoporous shell layer of HPP endows great potential in drug delivery. A preliminary trial was conducted to encapsulate DOX into HPP. By carefully regulating the experimental parameters, a drug loading efficiency of 60% was obtained (Additional file 1: Fig. S10). Considering the synergistic effect of PTT and chemotherapy, we envisage a bright and promising future of HPP in cancer therapy.

## Conclusions

In this contribution, HPP nanostructure with uniform morphology and large inner cavity was developed through a facile approach by applying SiO_2_ nanoparticles as sacrificial templates. Owing to the abundant sp^2^ domains in nanocarbon materials, HPP can efficiently absorb NIR light and generate thermal energy. A remarkable high HCE of 45.1% was obtained under the irradiation of NIR-II 1064 nm laser, which is among the best of previously reported NIR-II PTAs. With the surface modification of PEG-g-PEI, HPP exhibited negligible bio-toxicity with working concentration. Importantly, cancer cells in vitro and in vivo can be efficiently killed by photothermal effects, which were realized by a low concentration (40 µg/mL) of HPP in PBS solution under the irradiation of a 1064 nm laser with a safe power density of 0.6 W/cm^2^. PTT as an emerging therapeutic modality exhibits a precise and efficient nature over conventional cancer treatment. HPP, fabricated in this work, possesses a reliable and powerful photothermal effect under NIR-II laser and limited bio-toxicity, is a great candidate for future PTT in cancer therapy. In addition, the inner cavity of HPP may also hold great promise for drug delivery and combined chemo-photothermal therapy.

## Supplementary Information

**Additional file 1.** Zeta potential of nanoparticle samples, photothermal responses of HPP, TEM images of HPP after laser treatment, bio-safety evaluation, and drug loading capacity of HPP.
