# Supplementary material for: NIR-II-activated biocompatible hollow nanocarbons for cancer photothermal therapy
Source: J Nanobiotechnology. 2021 May 13;19:137. doi: 10.1186/s12951-021-00884-7 (PMC8120736; doi:10.1186/s12951-021-00884-7)
Supplement: Supplementary file 1 — Additional file 1. Zeta potential of nanoparticle samples, photothermal responses of HPP, TEM images of HPP after laser treatment, bio-safety evaluation, and drug loading capacity of HPP. [file 12951_2021_884_MOESM1_ESM.docx]

**Supporting Information**

**NIR-II-Activated Biocompatible Hollow Nanocarbons for Cancer Photothermal Therapy**

Zhourui Xu^1,#^, Yinling Zhang^1,#^, Weixiao Zhou^1,#^, Lijian Wang^2^, Gaixia Xu^1^, Mingze Ma^1^, Fenghua Liu^3^, Zan Wang^3^, Yucheng Wang^4^, Tiantian Kong^1^, Binyuan Zhao^2,*^, Weiping Wu^3,*^, Chengbin Yang^1,*^

^1^Guangdong Key Laboratory for Biomedical Measurements and Ultrasound Imaging, School of Biomedical Engineering, Health Science Center, Shenzhen University, Shenzhen, 518060, China.

^2^State Key Laboratory of Metal Matrix Composites, School of Materials Science and Engineering, Shanghai Jiao Tong University, Shanghai, 200240, China.

^3^Laboratory of Thin Film Optics, Shanghai Institute of Optics and Fine Mechanics, Chinese Academy of Sciences, Shanghai, 201800, China.

^4^School of Physics and Optoelectronic Engineering, Xidian University, Xi’an 710071, China

* Correspondence: [cbyang@szu.edu.cn](mailto:cbyang@szu.edu.cn); [byzhao@sjtu.edu.cn](mailto:byzhao@sjtu.edu.cn); [wuwp@siom.ac.cn](mailto:wuwp@siom.ac.cn)

# Zhourui Xu, Yinling Zhang, and Weixiao Zhou contributed equally to this work


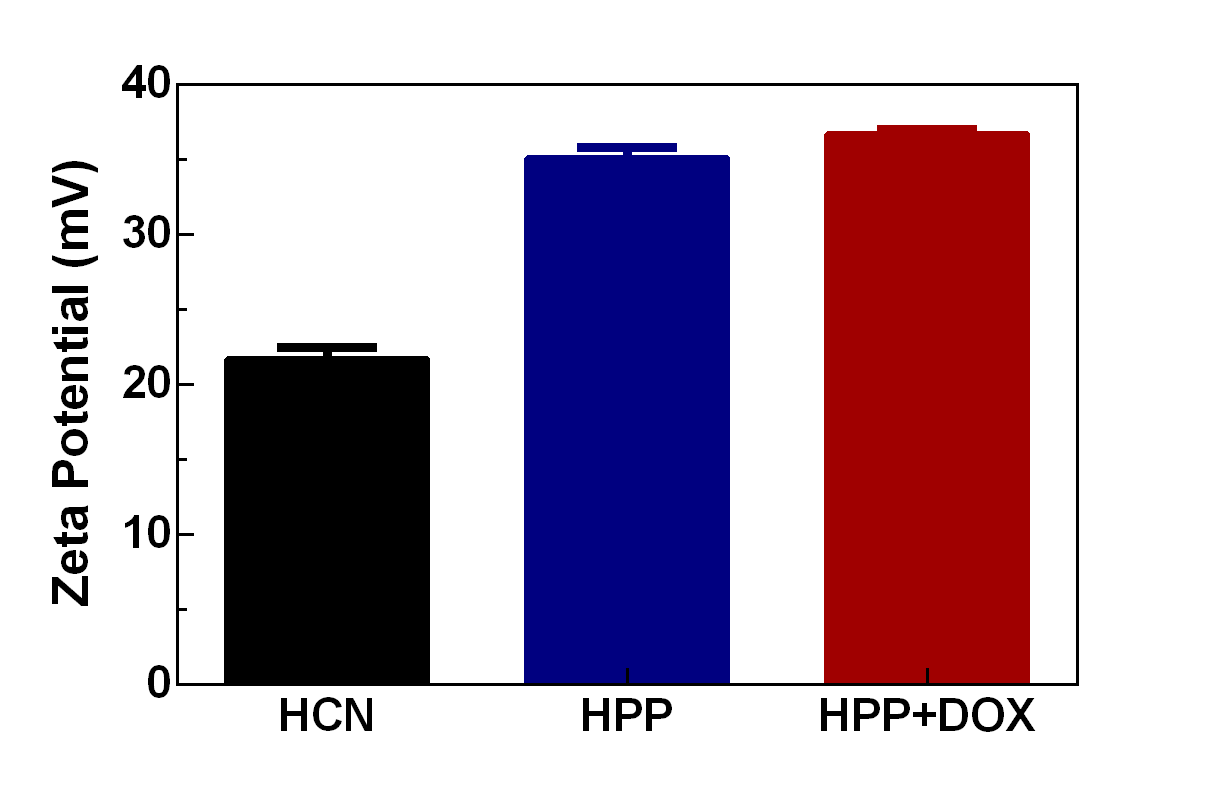


**Fig. S1** Zeta potential of hollow carbon nanosphere (HCN), HPP, doxorubicin (DOX) and DOX-loaded HPP.


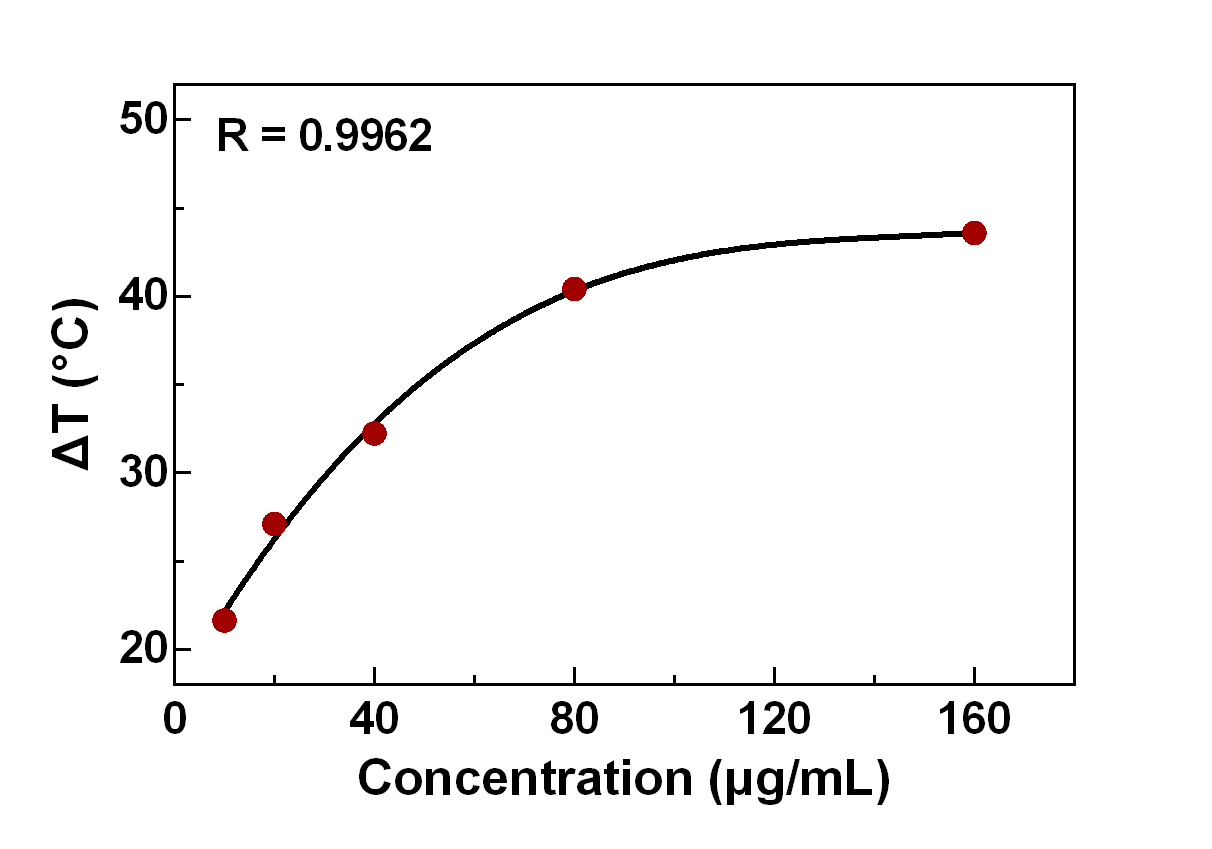


**Fig. S2** Relationship between final temperature changes at the end of laser irradiation and the concentrations of HPP.


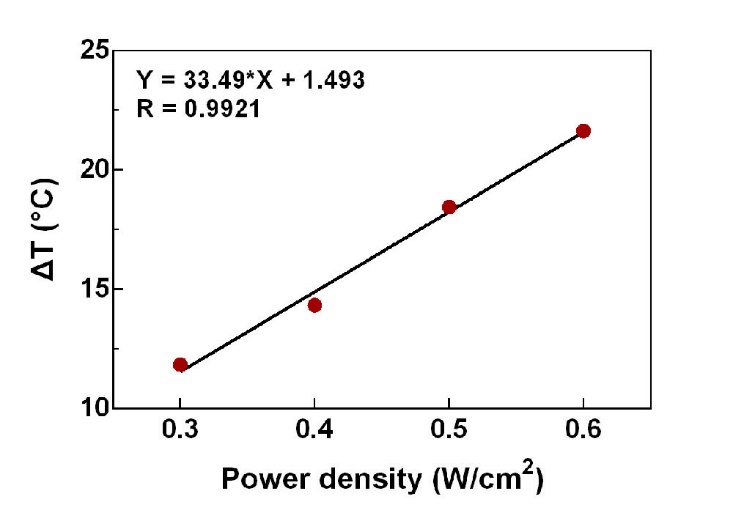


**Fig. S3** Relationship between final temperature changes at the end of laser irradiation and the power density of the 1064 nm laser.


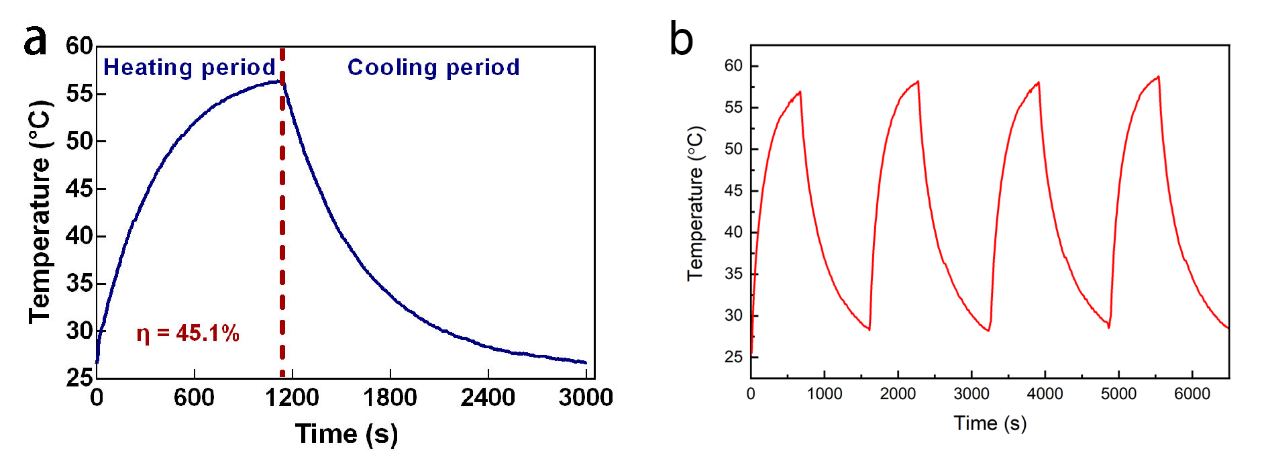


**Fig. S4** **a** Temperature change with time up under laser irradiation and the cooling process when the laser is switched off. The HPP dispersion was firstly irradiated by 1064 nm laser (0.6 W/cm^2^) to the saturated temperature, followed by natural cooling with laser turned off. **b P**hotostability of HPP evaluated by performing the lasering and cooling cycle several times.


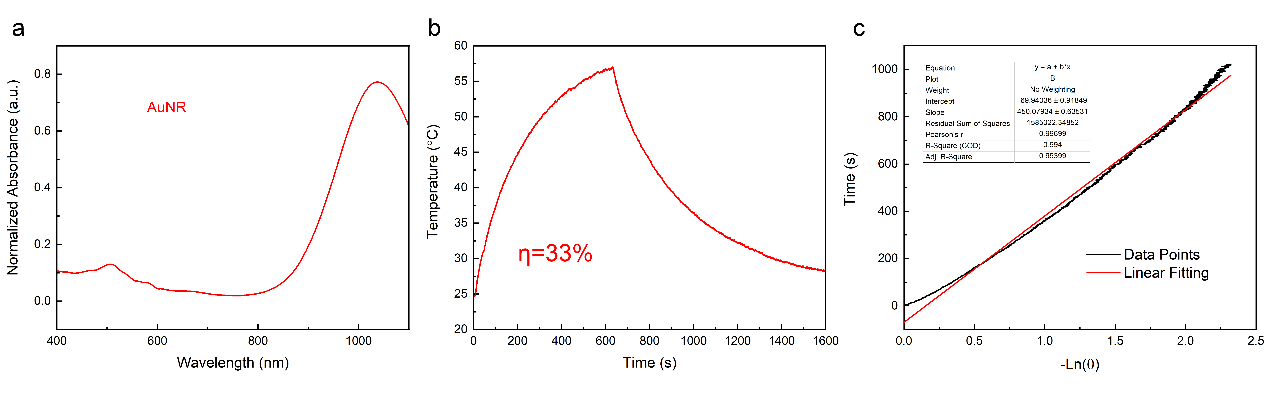


**Fig. S5** **a** Visible-Near Infrared absorbance spectra of gold nanorods suspension. **b** Temperature change with time up under laser irradiation and the cooling process when the laser is switched off. (laser: 1064 nm; Power density: 0.6 W/cm^2^). **c** Linear time data versus -ln(θ) obtained from the cooling period of Fig. S5b.


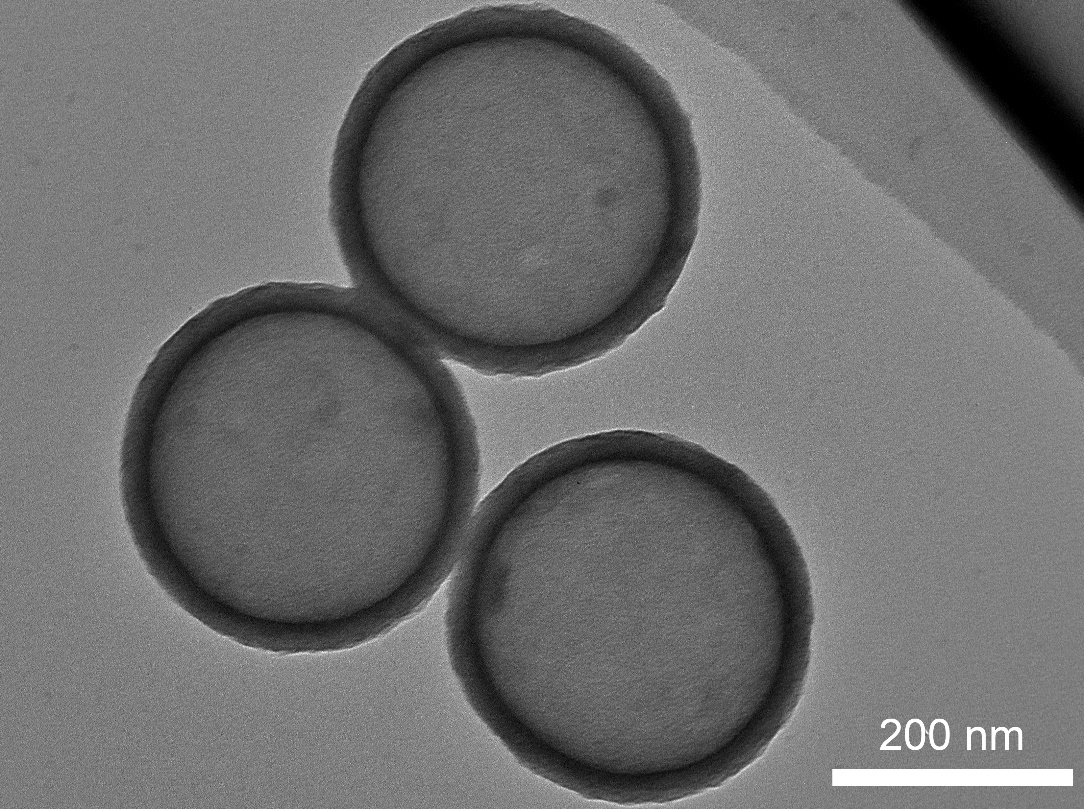


**Fig. S6** TEM image of HPP after four lasering and cooling cycles (laser: 1064 nm; Power density: 0.6 W/cm^2^).


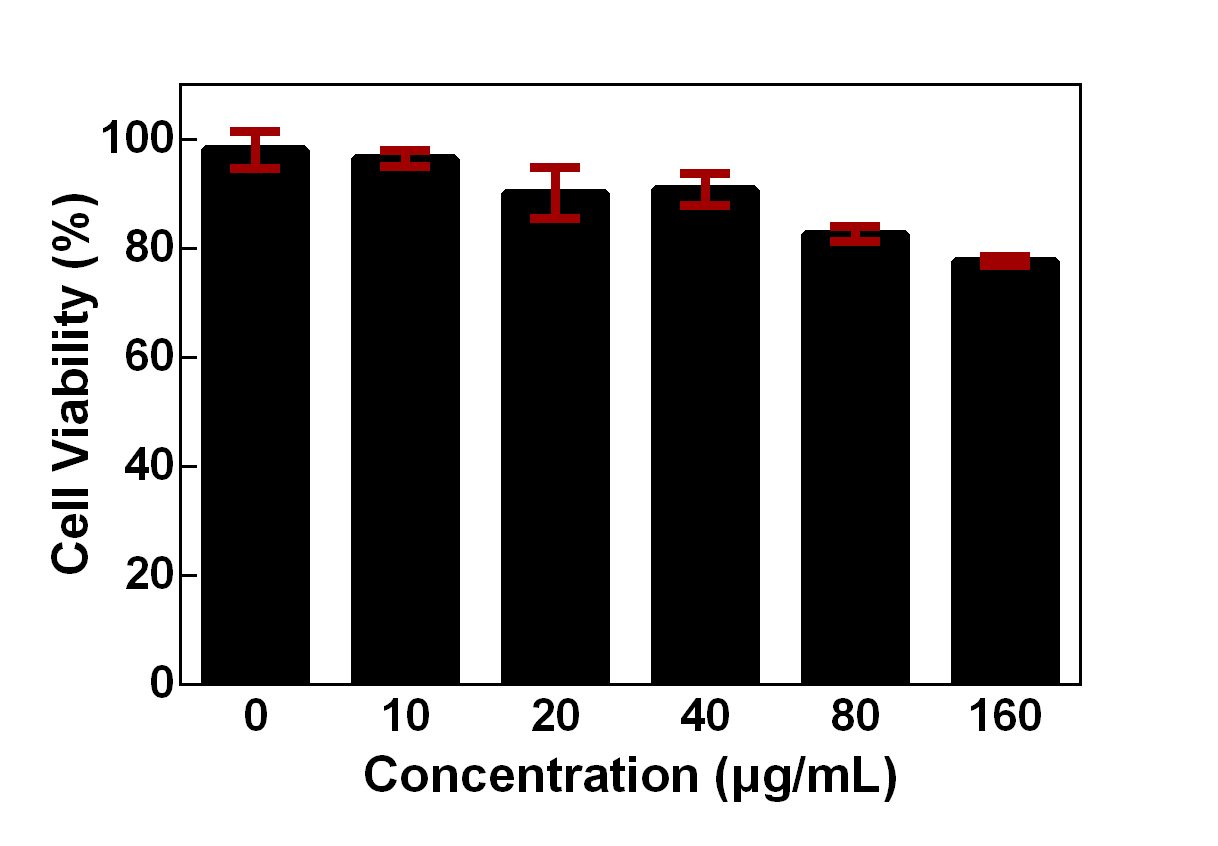


**Fig. S7** After incubation with different concentrations of HPP for 24 hours, the cell viability of MCF-7 cells was evaluated by MTT assay


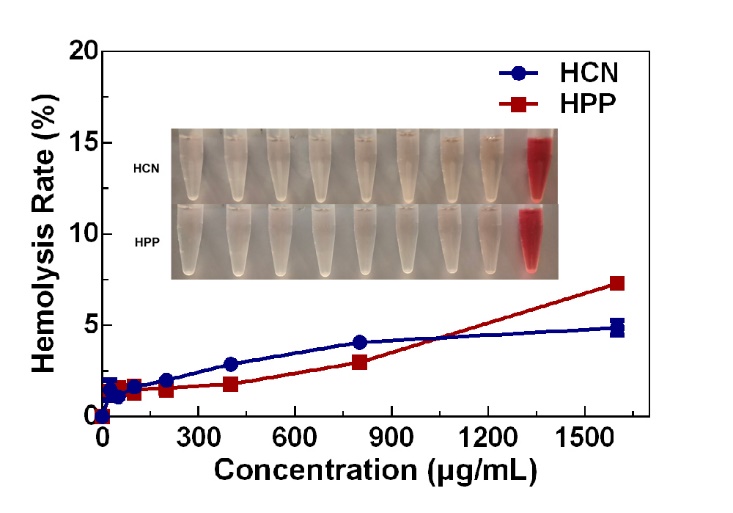


**Fig. S8** Hemolysis assay of naked HCN and HPP. Red blood cells were incubated with various concentrations of HCN and HPP. The mixtures after kept standing for 3 h were centrifuged to detect the released hemoglobin in the supernatant visually. The absorption of pelleted RBC supernatant was measured by a plate reader. Results are expressed as percentage of total RBC lysis as determined using the DI water as positive control. Data were presented as mean ±SD, n=3.


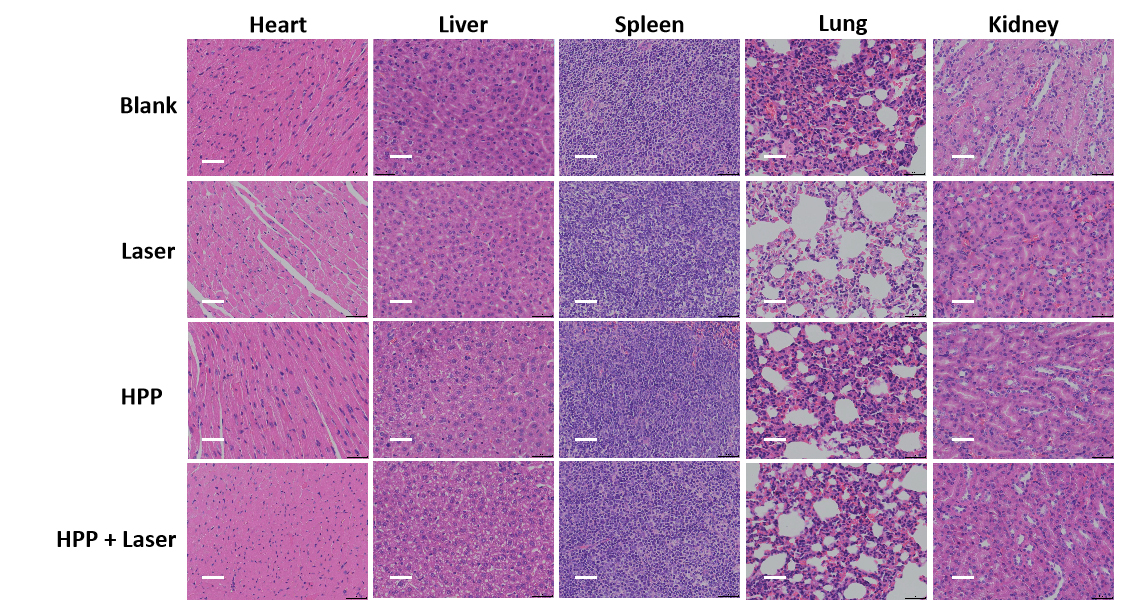


**Fig. S9** Hematoxylin and eosin (H&E) images of the main organs (heart, liver, spleen, lung, and kidney) collected from treated mice. Scar bar is 100 μm.


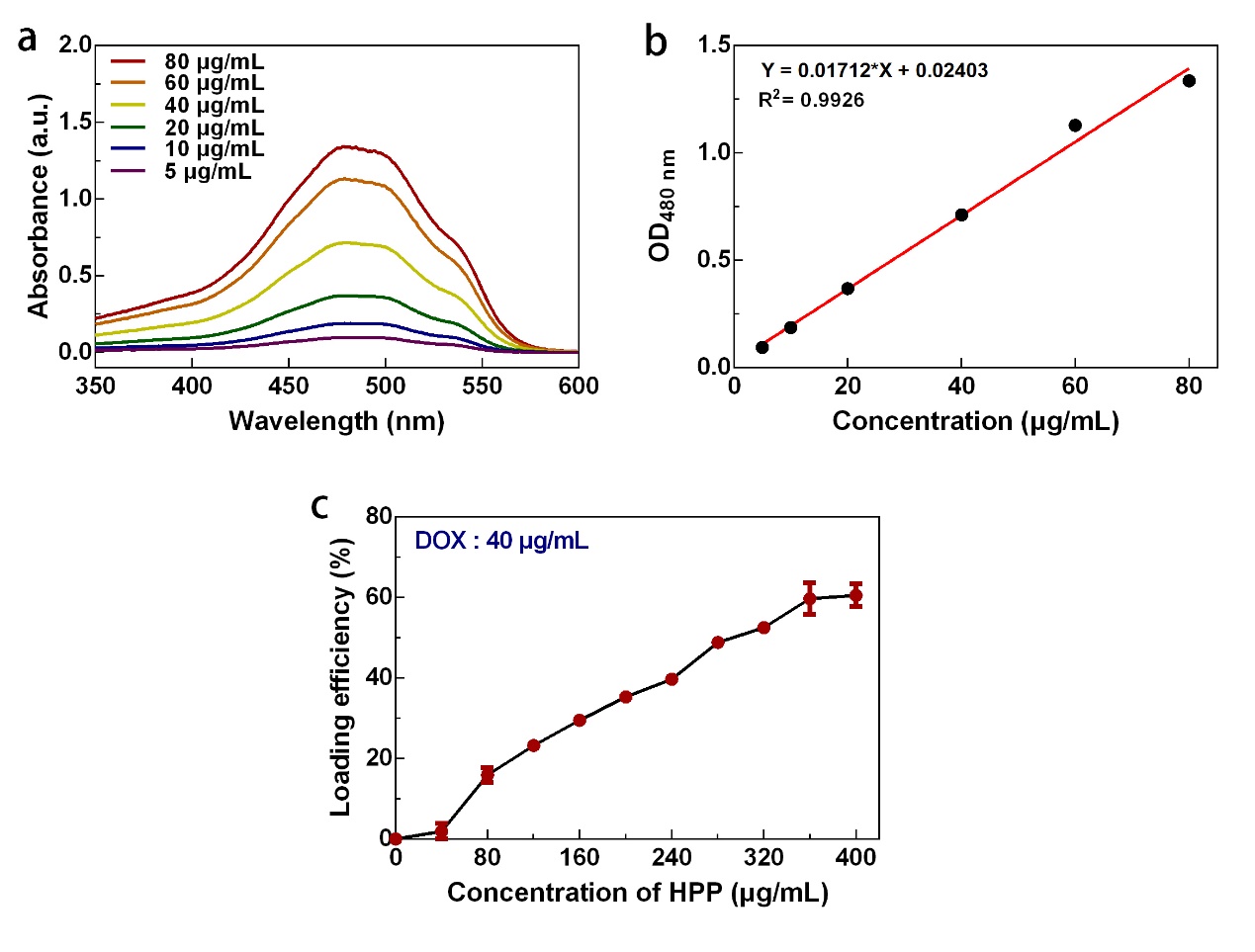


**Fig. S10** Large inner cavity of HPP can also be utilized as drug reservoirs. DOX was used as a model drug to evaluate the drug loading efficiency of HPP. **a** Absorbance of DOX with varied concentrations was firstly measured. **b** Standard curve of DOX contrations with the absorbance . **c** Relationship between drug loading efficiency and the concentration of HPP.
